# Air Quality Assessment by the Determination of Trace Elements in Lichens (*Xanthoria calcicola*) in an Industrial Area (Sicily, Italy)

**DOI:** 10.3390/ijerph19159746

**Published:** 2022-08-08

**Authors:** Daniela Varrica, Federica Lo Medico, Maria Grazia Alaimo

**Affiliations:** Dipartimento Scienze della Terra e del Mare (DiSTeM), Università di Palermo, Via Archirafi 22, 90123 Palermo, Italy

**Keywords:** trace elements, industrial area, atmospheric pollution, biomonitoring

## Abstract

This study provides data on variation in the content of metals and metalloids measured in the lichens (*Xanthoria calcicola* Oxner) collected in the Syracusan petrochemical complex (Sicily, Italy) which is considered one of the largest in Europe. Concentrations of eighteen trace elements measured in the lichens that were collected from 49 different points were analyzed using an inductively coupled plasma (ICP-MS) device. The concentrations of the typical elements of industrial emissions (As, Cr, Ni, and V) highlight the environmental criticality that exists in the study area. The interpretation of the data in terms of multi-element statistical analysis (FA) and enrichment factor (EFs) proved to be particularly useful in identifying several sources that contribute to the presence of trace elements in the atmospheric particulate between anthropogenic emissions and geogenic emissions. The results of this study reveal the versatility of the lichen species *Xanthoria calcicola* Oxner in the search for trace elements in highly anthropized environments, so the approach followed in this study can also be applied to other industrial contexts.

## 1. Introduction

Trace elements emitted into the atmosphere from various anthropogenic sources are significant due to their threat to the environment and human health. Extensive research has been conducted in recent decades to determine the distribution, sources, and toxicity of atmospheric trace elements in population centers, and industrial and economic areas around the world [1,2,3,4]. Metal pollutants are toxic and are considered important due to their nature of rapid dispersion, persistence, and bioaccumulation [5,6]. Several kinds of research have revealed that exposure to high concentrations of metal pollutants causes damage to the central and peripheral nervous systems, lungs, kidneys, and liver and can further lead to death. However, prolonged exposure to even low concentrations of metal pollutants irritates the nose and throat, and can lead to cough, dyspnea, and asthma [7,8].

The monitoring of environmental quality by cosmopolitan organisms has developed considerably in recent decades [9,10,11]. The types of organisms used can react, respond, or adapt to changes in environmental quality, both as an individual and as a community. Over the past 50 years, biomonitoring studies using lichens as a biological indicator have increased and expanded in terms of various parameters, monitoring techniques, and sampling areas [12,13,14,15,16,17,18,19,20]. Since then, lichens have been the most studied biological indicator [13,21] and have been defined as “permanent control systems” for assessing air pollution. Biomonitoring with lichens has several advantages over conventional techniques, such as low cost, easy sampling, and the ability to monitor large areas [22]. Lichens are organisms formed by a symbiotic relationship between a fungus (mycobiont) and one or more photosynthetic partners, algae, or cyanobacteria (photobiont). Lichens are slow-growing and long-lived organisms and depend on nutrients, from wet and dry atmospheric deposition for their growth and metabolism [23]. Their morphology does not change seasonally. Lichens can accumulate and retain trace elements at levels above their physiological needs [24] without showing morphological changes. Some lichen species found around industrial areas, power plants, and highways can be used to detect heavy metal pollution in the air. Since lichens accumulate elements from the atmosphere directly in their tissues, there are several studies on the evaluation of heavy metal pollution in the air through lichens [25,26,27].

Over time, the concentrations of trace elements in the lichen thallus reach an equilibrium with the average levels of air pollution [28], and the chemical composition of the lichens reflects the availability of trace elements in the environment [29]. In this way, the accumulation of air pollutants in lichen thalli can be used to evaluate the patterns of spatial and temporal deposition [30].

This study aims to examine the spatial distribution of the concentrations of elements through the analysis of lichens in highly anthropized environments of the Syracusan petrochemical complex, defined by the Italian government as an area at “high risk of environmental crisis”. Therefore, we also aimed to (1) evaluate the atmospheric deposition of trace elements at different distances and directions from the industrial plants; (2) identify common sources of trace elements in the area; (3) distinguish natural from anthropogenic sources of trace elements through multivariate statistical analysis.

The results presented here belong to a larger research project on the dispersion of heavy metals and metalloids in various matrices (vegetation, soil, atmospheric particulate, and groundwater) in the industrial districts of Sicily (Italy) and the definition of regional background and baseline levels.

## 2. Materials and Methods

### 2.1. Description of the Study Area

The study area falls within the Syracusan petrochemical complex which is considered one of the largest in Europe. The main activity of the settlement is the refining of oil, the transformation of its derivatives, and energy production companies. This industrialized coastal area of eastern Sicily includes the municipalities of Augusta (about 36,000 inhabitants), Priolo Gargallo (about 11,400 inhabitants), and Melilli (about 13,000 inhabitants), covering an area of about 300 km^2^ and reaching the gates of Syracuse.

Industrial activities began in the 1950s and developed rapidly until the 1980s, making the site the most important hub in Europe. Subsequently, some industries have closed, while others are still active. Since the 1980s, several epidemiological studies have found a strong incidence of lung tumors in the male gender but, above all, a high incidence of births of malformed children is reported [31,32]. Only in 1990 was this area defined as an area at “elevated risk of environmental crisis” by the Italian Government (Italian Law n. 349/1986, [33]). Since 1998, Augusta, Priolo Gargallo, and Melilli were included among the 57 Italian Polluted Sites (IPSs) of national interest for environmental remediation (Italian Law n. 426/1998, [34]).

**Figure 1:**
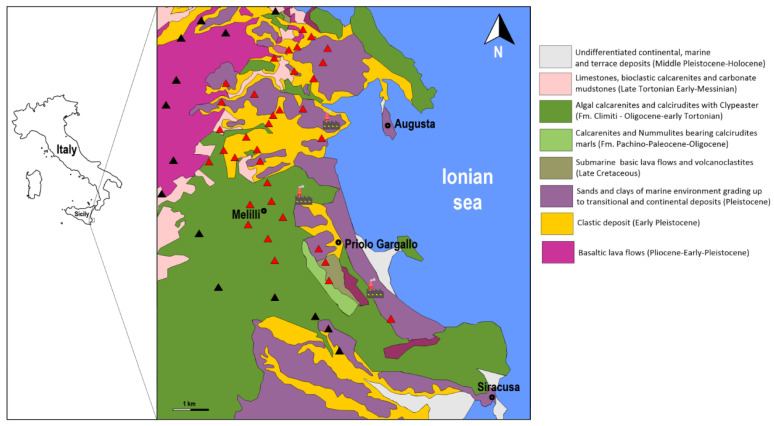
Geological sketch map of the study area with the location of the sampling sites (modified from [35]). Control samples (SUBIND) collected in rural environment (black triangle); lichen samples (IND) collected near the Syracusan petrochemical complex (red triangle).

The outcropping rocks in the study area are mainly of sedimentary origin characterized by alluvial and fluvial deposits together with coarse sands, and limestone dating back to the Upper Cretaceous, with sporadic outcrop of basaltic rocks [35] (Figure 1).

**Figure 2:**
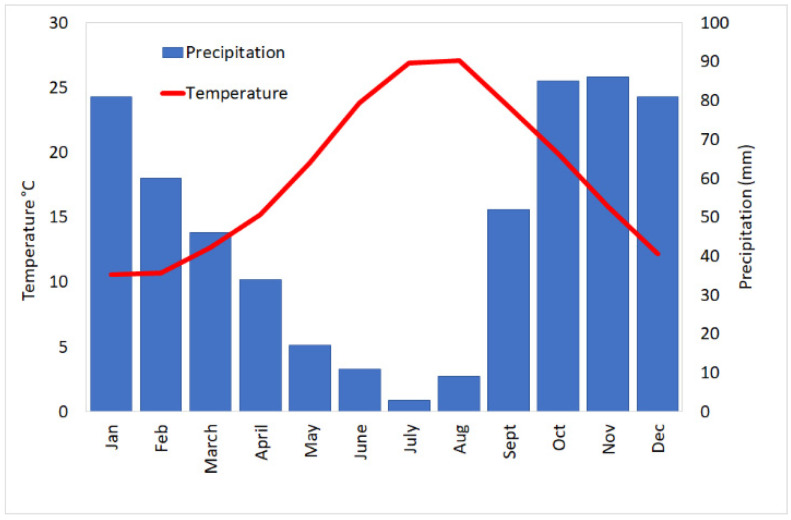
Weather data from meteorological station of Augusta. Mean monthly precipitation (mm) and mean monthly temperature (°C).

The climate classification of the study area is Csa type (Mediterranean climate with hot summer) as established by Köppen and Geiger. The study area is characterized by a period of greater rainfall in the winter months than in the summer (Figure 2). The average annual temperature is 17.8 °C. In general, the predominant winds in the area under consideration are those coming from the southern quadrants; among these the most representative is the sirocco. Due to the different heating of the sea and the land, land and sea breeze is determined: the first occurs during the night and the second during the day [36].

### 2.2. Sampling and Analytical Method

A total of 49 composite samples of lichens, *Xanthoria calcicola* Oxner, were collected in November 2019 in the Syracusan petrochemical complex and its surroundings, from an irregular sample grid. The locations depended on the availability of lichens randomly distributed near the petrochemical plant (IND-35 samples) and distant from the polluting sources that can be considered as control samples (SUBIND-14 samples). Samples were taken from tree trunks and rocky substrate by scraping with a plastic spatula and carried to the laboratory in a plastic bag.

The non-washed samples were dried for 24 h at 40 °C and then ground to a fine powder using an agate mortar. Powdered lichens (~0.500 g) were digested with a mixture of 5 mL of 65% HNO_3_ (Suprapur, Merck, Germany) and 2 mL of 30% H_2_O_2_ (Suprapur, Sigma Aldrich, St. Louis, MO, USA). The digest was diluted to 50 mL using deionized water. In each sample, 16 trace elements (Al, As, Ba, Co, Cr, Cu, Fe, Mn, Mo, Pb, Rb, Sb, Sr, U, V, Zn) were determined using an inductive coupled plasma mass spectrometer (Elan 6100 DRC-e, Perkin Elmer, Waltham, MA, USA) at the Dept. Scienze della Terra e del Mare, University of Palermo, after the addition of Re–Sc–Y as an internal standard. For As, Cr, Fe, and V the ICP-MS was operated in DRC (Dynamic Reaction Cell) mode with CH_4_ as reaction gas. All standard solutions were prepared with 18 MΩ·cm deionized water, the ICP Multi-Element Standard Solutions XXI CertiPUR (Merck, Kenilworth, NJ, USA), and the Mo and Sb CertiPUR standards (Merck). Calibration curves ranging from 0.05 μg L^−1^ to 500 μg L^−1^ were constructed. To minimize matrix effects, the standard addition technique was used for all metal determinations. Sample blanks were also analyzed, and the operational limit of detection (LOD) for each element was calculated as 3 times the standard deviation of the analyte concentration in blank samples. To validate the analytical procedure, the standard reference material NIST SRM 1515 Apple Leaves was analyzed for corresponding elements. The metal recovery rates resulted in good agreement with the certified concentrations, ranging between 89% and 102%. 

Analytical precision, estimated from triplicate analyses of every tenth sample, was in the range of 3–11% for all analyzed elements.

### 2.3. Statistical Analysis and Spatial Distribution

Data were analyzed statistically by the STATISTICA program (Tulsa, OK, USA), StatSoft version 6.0. All the tests in this study were considered significant at *p* < 0.05. Shapiro-Wilk test, with a level of significance set at *p* < 0.05, was used to verify the normality of data distribution. The non-parametric Mann-Whitney test at *p* < 0.05 was also used to verify the statistical significance of observed differences between IND and SUBIND samples. The Spearman correlation matrix was used to measure the association between variables in terms of rank. To identify possible common sources of pollutants, we evaluated the relationships between trace elements by factor analyses.

To spatially represent pollutant patterns, we elaborated distribution maps for the same trace element using the program SURFER v. 8.05 (Golden, CO, USA). The maps were based on a grid created by the Kriging interpolation method [37].

### 2.4. Geochemical Indices for Evaluating Trace Element Pollution

The *Contamination Factor* (CF) is called the pollution index, which expresses the quotient obtained by dividing the metal concentration in the investigated site by the background values [38]. The CF was calculated using Equation (1:)

CF = C_m_/C_b_ (1)

where C_m_ is the concentration of an element measured in lichen species and C_b_ is the background level of the element in the lichen. The lichen sample collected distant from the polluting sources can be considered for extracting the background values of metals and metalloids. The contamination factor of each metal was classified as follows [39,40]:

CF 0–1 No contamination
CF > 1–2 Suspected contamination
CF > 2–3.5 Slight contamination
CF > 3.5–8 Moderate contamination
CF > 8–27 Severe contamination
CF > 27 Extreme contamination

The *Pollution Load Index* (PLI) originally proposed by Tomlinson et al. (1980) was also used to evaluate the overall air pollution load, as follows: PLI = (CF1 × CF2 × CF3 × CF4 ×…× CFn)^1/n^, where CF is a contamination factor and n is number of studied elements. A PLI value less than 0. 9 indicates an unpolluted area, a PLI approaching 1 indicates an air pollution load close to the background level [41], and a value higher than 1 indicates low pollution (1.1 < PLI < 1.5), moderate pollution (1.5 ≤ PLI < 2.0), or very high pollution (PLI ≥ 2.5). 

The *Ecological risk index* (ERI) is used for quantitatively expressing the potential ecological risk of investigated metals and is calculated as shown in Equation (2) [38,42]:

ERI = T_ri_ × CF (2)

where T_ri_ is the toxic response factor for the metals/metalloids and CF is the contamination factor. The T_ri_ values of metals/metalloids (As = 10, Cr = 2, Cu = 5, Ni = 5, Pb = 5, V = 2, Zn = 1) were reported by Hakanson [38], Qing et al. [43], and Wu et al. [44]. The ERI values can be classified as per the following:

ERI < 5 Low risk
5 ≤ ERI < 10 Moderate risk
10 ≤ ERI < 20 Considerable risk
20 ≤ ERI < 40 High risk
ERI ≥ 40 Very high risk

Although risk indices were initially used as a diagnostic tool to control water pollution, in recent years they have also been widely used to assess the quality of sediments and soils [45], and recently in biomonitoring [46].

The Enrichment Factor (EF) is defined as EF = (X/Ref)_sample_/(X/Ref)_soil_, where X is the element of interest and Ref is the reference element; the index sample indicates the analyzed sample (lichen, airborne particulate matter, vegetation, soil, etc.) whereas the soil index is relative to the average concentration of X and Ref in the local parent material. There are no universal fixed rules for the choice of reference element, except that it must be immobile and almost exclusively of crustal origin. In calculating the enrichment factor, Al, Fe, Sc, or Ti are the most used elements as reference. In this study, we used aluminum as a reference element based on these considerations: (1) it is of high natural abundance, (2) it is easily determined by conventional techniques, and (3) it may be assumed as derived wholly from soil sources, and (4) has a limited metabolic value in lichens [47].

## 3. Results and Discussion

### 3.1. Descriptive Statistics

The main statistical parameters of the analyzed elements in lichens samples collected near the Syracusan petrochemical complex (IND) and its surroundings (SUBIND) are reported in Table 1, which also shows the data grouped as a single dataset (TOT). Most of the elements analyzed, based on the test Shapiro-Wilk test (*p* < 0.05) show asymmetric distribution, and only Al and Ba show a normal distribution. In the total database, median concentrations of the most abundant elements ranged from 8200 μg g^−1^ (Al) to 600 μg g^−1^ (Ti) with the following order of abundance: Al > Fe > Ti. The elements Mn > Zn > Ba > V > Sr > Pb > Ni > Cr, Rb > Cu, according to the following order of abundance, showed median concentrations in the range of 85–14 μg g^−1^. The remaining elements showed a concentration below 3 μg g^−1^ (Co > As > Mo, Sb > U). 

The relations among the elements were evaluated by the Spearman correlation matrix. The elements showing significant correlation with each other have common sources and were exposed to similar atmospheric conditions. The correlations between the elements are provided in Table 2. 

A strong association between elements of crustal origin Al and Ti was identified with R = 0.91 (*p* < 0.05). Various elements of typical geogenic contributions such as Ba, Co, Rb, and Sr reveal good correlations (R = 0.65–0.72) highlighting the heterogeneity of the type of outcropping rocks. For Sr, it is not possible to exclude an anthropic contribution, considering the good correlations with Cr and Mn (R_Cr_ = 0.71; R_Mn_ = 0.67, *p* < 0.05). Furthermore, good correlations have also been found between Cr, Fe, and Mn (R_Cr-Fe_ = 0.66; R_Cr-Mn_ = 0.64; R_Mn-Fe_ = 0.63). The link between these elements may be crustal origin [48]. The Spearman correlation coefficients between V, Ni, Sb, Pb, and Zn were calculated within the range of 0.68–0.87 (*p* < 0.05). These elements are mostly emitted from anthropogenic activity such as fossil fuel combustion and refineries [48,49].

From the comparison of the median values in the two groups (IND-SUBIND) reported in Table 1, it is observed that the abundance orders in the total database are generally respected except for Zn > Mn, Pb > Sr, and Rb > Cr, Cu in the IND database. Furthermore, from the comparison of medians between groups (IND-SUBIND), concentration differences are observed in some trace elements (Al, As, Fe, Mn, Ni, Pb, Ti, V, and Zn). A non-parametric Mann-Whitney test (at significance level *p* < 0.05) was applied to verify the significant differences in metal and metalloid concentrations in lichen samples between IND and SUBIND. The results reported in Table 1 show that Al, As, Cr, Fe, Mn, Ni, Pb, Sb, Ti, V, and Zn were statistically significant differences. 

**Figure 3:**
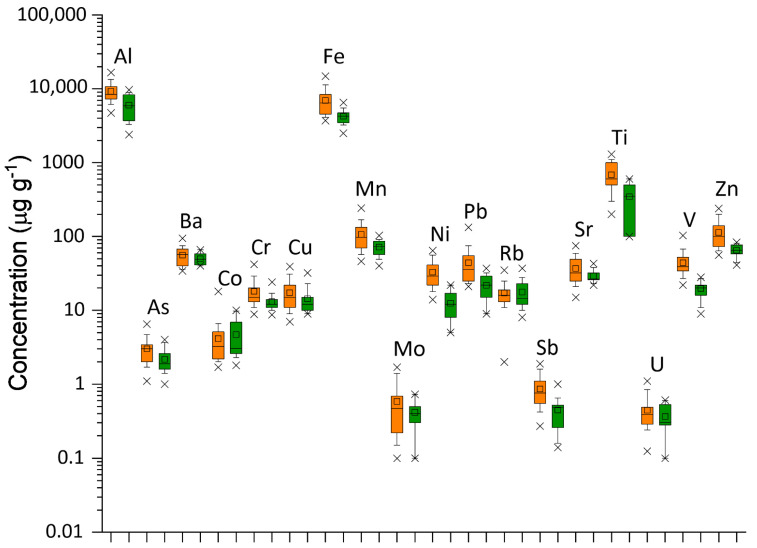
Box plot of chemical elements in the analyzed lichen samples. Boxes delineate the interquartile range (25–75%) with the indication of the median (dark line); a small square inside the box marks the mean value; whiskers indicate the 10–90% range; points outside the box are minimum and maximum values. Data are given in μg g^−1^. Lichen samples IND (orange box); Lichen samples SUBIND (green box).

Figure 3 shows the metal profiles of chemical elements in lichen group_IND_ and group_SUBIND_. Al, Co, Fe, Rb, Sr, Ti, and U are considered typical crustal elements, even if a minority anthropogenic component cannot be excluded for iron and titanium. The elements of typical anthropogenic sources (As, Cr, Cu, Mn, Mo, Ni, Pb, Sb, V, and Zn) show higher concentrations in industrial sites than in control samples. 

The statically different elements were used in Factor Analysis (FA) to identify relationships between variables and possible sources of air pollution. The FA model used in this study was applied to elemental raw data. The input variables were the concentrations of 11 selected elements in lichen samples. The raw calculated factor loading coefficients were rotated by Kaiser’s varimax rotation scheme [50]. 

The obtained factor loadings for the three-factor model, together with communalities, are given in Table 3. The loadings indicate the influence a variable has on the formation of each factor; communality indicates the total variance of a variable explained by the combination of the three common factors. FA indicates that approximately 79% of the variance may be explained by the first three factors. Factor 1, showing high positive loadings on elements Ni, Pb, Sb, V, and Zn, accounts for 32% of the total variance in the database. These are typical elements associated with fuel burning; this factor is named the “petrochemical factor”. 

The dominant elements in Factor 2 are As, Cr, Fe, and Mn (24% of total variance). This factor is mainly attributed to the influence of different manufacturing processes present in the investigated area. The profile of Factor 3, which accounts for a further 23% of the remaining variance, is determined by elements such as Al, and Ti. Due to the dominance of elements of typical crustal origin, this factor confirms the fundamental role of resuspension of soil particles in determining the total quantity of particulate matter on lichen samples. 

**Figure 4:**
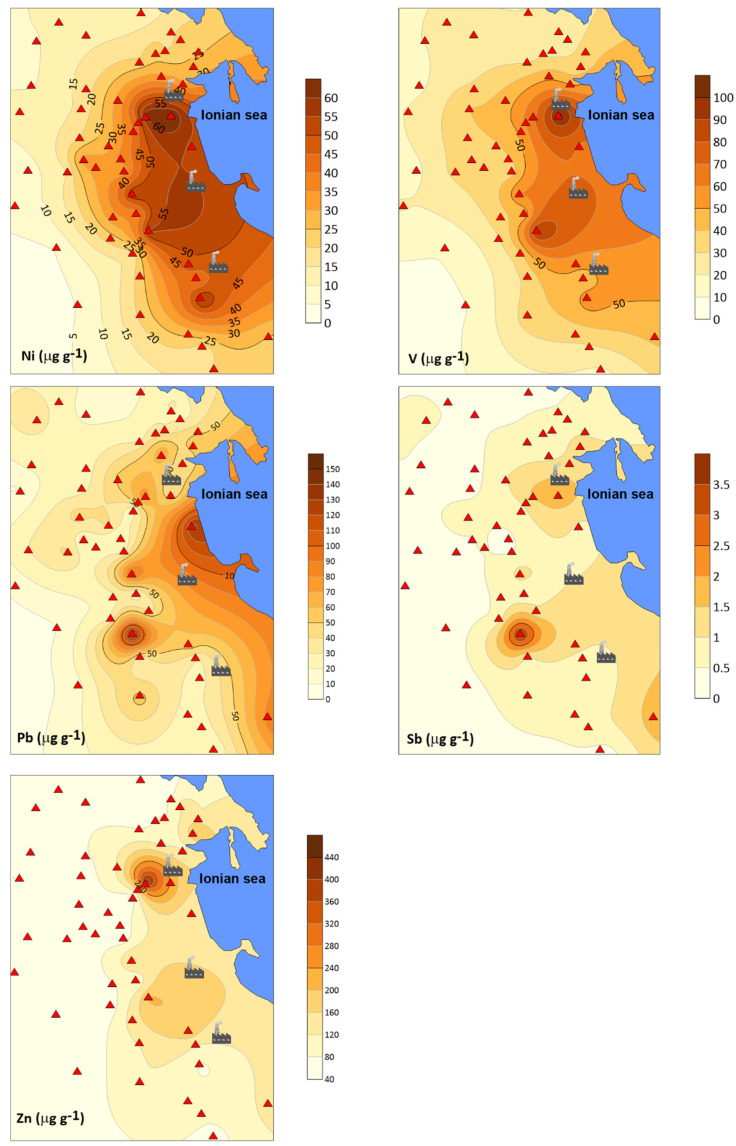
Distribution maps of some trace elements in lichens from the survey area. Concentrations are given in μg g^−1^.

Figure 4 shows the spatial variations of accumulation of some trace elements representative of the main factor extracted from the FA. The highest concentrations of Ni, Pb, V, and Zn are generally detected in samples collected near petrochemical plants.

### 3.2. Enrichment Factor and Geochemical Indices

**Figure 5:**
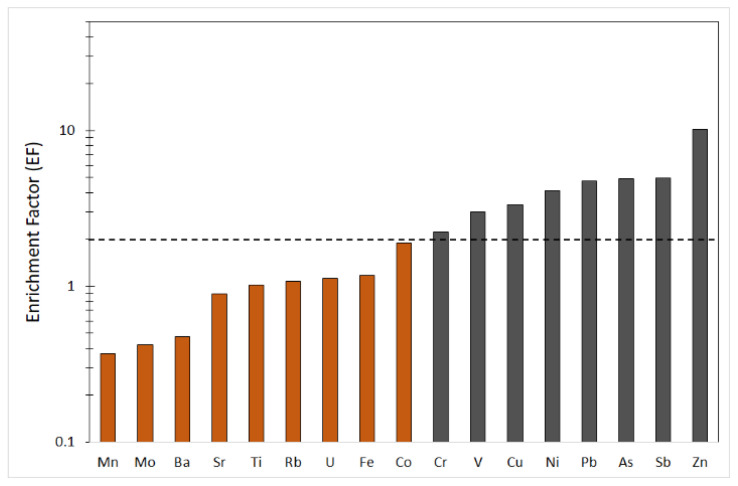
Average enrichment factors (EFs) for the analyzed elements in lichen samples from the survey area. The dashed line indicates the boundary between enriched and non-enriched elements.

A serious problem in the interpretation of biogeochemical data is being able to distinguish between the origin of elements that are derived from soil and those arising from anthropogenic sources. To better understand the datasets, enrichment factors (EFs) for all elements were calculated, to differentiate natural and anthropogenic inputs in lichens. On the basis of the considerations by Hernandez et al. [51], EF values ranging between 0.5 and 2 can be considered in the range of natural variability, whereas ratios greater than 2 indicate some enrichment corresponding mainly to anthropogenic inputs. The calculated EF values between 0.5 and 2 are indicative of a geogenic input. Trace elements such as As, Cr, Cu, Ni, Pb, Sb, V and Zn had EF > 2, indicating a certain enrichment corresponding mainly to anthropogenic inputs (Figure 5). To know the level of distribution and absorption of heavy metals, the contamination factor (CF) was calculated for the trace elements that were found to be enriched by the EF. The selected elements are classified in the following sequence: Ni > V>Pb > Sb > Zn > As, Cr, Cu (Table 4), indicating that these metals and metalloids contribute to slight pollution the study area. Using the same elements, the pollutant load index (PLI) of the studied area was calculated to equal 1.76. The obtained value indicates moderate overall trace element contamination in the study area. In terms of the ecological risk posed by metals, As, Ni and Pb represent a considerable risk. Furthermore, Cu and V impose a moderate ecological risk in the order of V < Cu. Both CF and ERI calculations suggested that the environmental state of the study area is primarily threatened by As, Ni, Pb, V, and Cu. These metals can derive from various anthropogenic activities but are mainly emitted by industrial plants. The CF and ERI values are reported in Table 4.

### 3.3. Pollution Assessment

**Figure 6:**
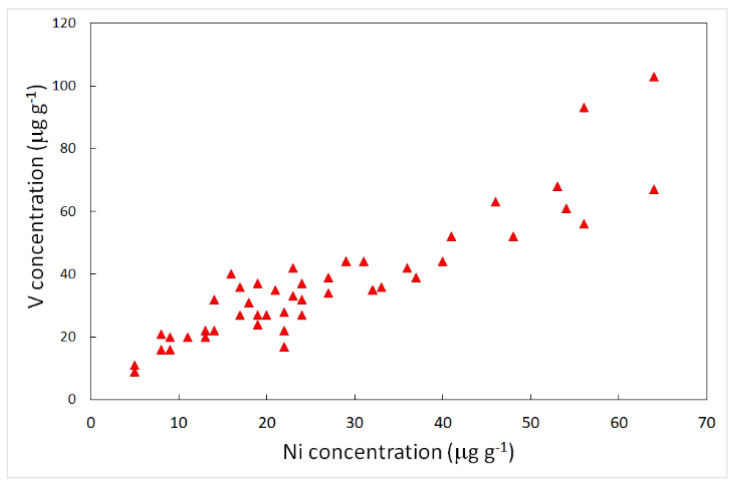
Relationships among Ni and V concentrations in lichen samples. Data are given in μg g^−1^.

Some elements traced by the factor analyses and enrichment factors are listed as potentially toxic to humans. These elements do not play an essential role, but several studies have emphasized that their presence in the human body can induce disease. The International Agency for Research on Cancer (IARC) has classified vanadium pentoxide as possibly carcinogenic to humans [52]. Although there is some evidence that vanadium is an essential nutrient, its functional role in humans has not been established [53]. It is well-known that the most important source of vanadium in the atmosphere is fossil fuel combustion [54] In this study, the highest concentrations of vanadium in lichens were found in those samples collected close to the industrial zone, and this was also true for a nickel. The analyses revealed high contents of nickel, corresponding to high contents of vanadium, with a correlation coefficient of 0.87 (Figure 6). 

The industrial origin is further supported by the comparison of the V/Ni ratio in pet-coke: 1.4 [55], with the same ratio calculated in the IND group (1.41) compared to the average value observed in SUBIND lichens (1.69). Human exposure to high nickel concentrations in the environment may cause a variety of pathological effects [56,57,58]. Accumulation of nickel and nickel compounds in the body through chronic exposure may be responsible for a variety of adverse effects on the health of human beings, such as lung fibrosis, kidney and cardiovascular diseases, and cancer of the respiratory tract [59]. A high incidence of nasal and lung cancer in workers exposed to nickel and nickel compounds was observed [60]. Arsenic is widely distributed in the environment, especially in its organic and inorganic forms [61]. The main anthropogenic sources that play an important role in the dispersion of arsenic into the environment are mining, and the use of pesticides or petrochemical plants [62]. In 2012 the Agency for Toxic Substances and Disease Registry (ATSDR) ranked arsenic at the top of the list of dangerous substances [63]. In addition, the International Agency for Research on Cancer (IARC) classified arsenic as a human carcinogen. Antimony may be emitted into the environment through natural and anthropogenic sources. Human activities include smelting, fuel combustion, waste incineration, production of plastics and textiles, and brake wear [64]. US Environmental Protection Agency (USEPA) has listed antimony as a priority pollutant that can cause adverse effects on human health, with impacts on the skin, eyes, gastrointestinal tract, and respiratory system [65]. The IARC has classified antimony trioxide as a possible human carcinogen. The toxicity of Sb(III) is due to its greater affinity for red cells and thiol groups of cell constituents [66]. Lead is considered the most commonly studied element in various environmental compartments and its toxicity to humans has been known for over 2000 years. Human exposure to lead and its compounds can occur from various sources such as industrial processes, coal burning, ceramics, etc., although the main source of lead during the twentieth century was attributable to the use of gasoline containing lead, totally banned in 2002. Once absorbed by the body, lead accumulates in the blood and bones, as well as in organs such as the liver, kidneys, brain, and skin. Its negative health effects can be both acute and chronic because the human body fails to excrete lead. Accumulation of lead has been shown to affect the reproductive, hepatic, endocrine, immune, and gastrointestinal systems [67].

Anthropogenic elements identified in this research are compatible with various studies on different environmental matrices, both biotic and abiotic, carried out in the Syracusan petrochemical area. A study carried out by Mudu et al. [68] pointed out that the resident population in the last few decades has been exposed to various pollutants through multiple exposure scenarios involving inhalation and ingestion (drinking water, fish products, agricultural and zootechnical products), emphasizing that the concentrations determined exceeded the legislative threshold limits by several orders [68]. Nicotra et al. [69] showed the overcoming of Pb and Cd in various fish products (fish and shellfish). Di Bella et al. [70] show a strong accumulation of As and Pb in fish products; instead high concentrations of zinc have been found in beef and pork animal products. A study conducted on hair samples taken from adolescents residing in the same area showed an accumulation of Fe, V, and Zn [71].

To better understand the degree of pollution in the study area, the concentrations of the typical elements of industrial emissions (As, Cr, Ni, and V) detected in the samples of lichens taken near the industrial plant (IND) were compared with other industrial areas. The levels of As, Ni, and V determined in our samples were higher than those found in Huelva [72], San Paulo [73], Livorno [25], and Kocaeli [22]. The chromium level was consistent with concentrations measured in Huela and San Paulo [72,73], and higher than those found in Livorno [25]. This comparison highlights the environmental criticality that exists in the study area.

## 4. Conclusions

The results of this study reveal the versatility of the lichen species *Xanthoria calcicola* Oxner when searching for trace elements. Although it is impossible to establish a quantitative relationship between the concentration of a trace element in the lichen thallus and its concentration in the atmosphere, the data obtained show that the area around the Syracusan petrochemical complex is heavily affected by industrial emissions of metals and metalloids that can represent a potential danger for the local population. Although anemological conditions can favor the transport and dispersion of aerosol particles away from the source, the highest concentrations of pollutants were found near industrial plants. However, the variations in the content of a lichen species depend on various factors, such as the geological and geographical context of the growing environment and the presence of anthropogenic sources. The results of this study reveal high concentrations of Ni, Pb, V, As, and Cr compared to other European industrial situations. The interpretation of the data in terms of multivariate statistical analysis (FA) and enrichment factor (EFs) proved to be particularly useful. In particular, the Factor analysis has identified several sources that contribute to the presence of trace elements in the atmospheric particulate between anthropogenic emissions and geogenic emissions. By calculating the enrichment factor, the elements were classified into: geogenic elements, derived from the local crustal material (Ba, Sr, Co, Ti, Rb, U, Fe, Mo, Mn), and enriched elements (Zn, Sb, As, Pb, Ni, Cu, V, Cr), coming mainly from anthropogenic sources traceable in the various industrial sectors persisting in the area.

The calculation of the geochemical indices has established that the study area is certainly classified as an area where moderate pollution persists, which implies a broader study in the Syracusan area that also involves other environmental matrices. Lichen analysis turns out to be an interesting, useful, economical, and fast method for monitoring atmospheric deposition of trace elements. However, even if the lichens provide information on the long-term impact of even low levels of pollution, they do not accurately characterize the level of a certain pollutant or the total degree of pollution of a study area, therefore we suggest that the biomonitoring data are associated with continuous environmental monitoring of the chemical composition of the finest fraction of airborne particulate. In general, it is hoped that both geogenic and anthropogenic sources should be considered in the planning of environmental controls, since in municipalities close to industrial areas, in addition to emissions related to human activity, natural emissions are added.

## Figures and Tables

**Table 1:** Mean and median concentrations for 18 trace elements analyzed in lichen samples. TOT: total database; IND: samples near the industrial plant; SUBIND: control samples. Test U: Mann-Whitney test (*p* < 0.05). The level with statistical significance is indicated in italics.

		TOT		IND			SUBIND			Test U_IND-SUBIND_
	N	Mean	Median	N	Mean	Median	N	Mean	Median	*p*-Level
**Al**	49	8286	8200	35	9200	8400	14	6000	5900	*0.001*
**As**	49	2.8	2.6	35	3.0	3.0	14	2.2	1.9	*0.015*
**Ba**	39	54.5	54.0	27	55.8	57.0	12	50.0	49.0	0.495
**Co**	49	6.3	3.1	35	6.9	3.4	14	4.7	3.1	0.572
**Cr**	49	16.6	14.0	35	18.1	15.0	14	13.1	12.0	*0.009*
**Cu**	49	16.4	14.0	35	17.3	15.0	14	14.3	12.0	0.249
**Fe**	49	6172	5340	35	6939	6420	14	4255	4265	*0.0003*
**Mn**	49	96.0	81.0	35	105.8	97.0	14	71.4	73.0	*0.017*
**Mo**	38	0.6	0.5	27	0.6	0.5	11	0.4	0.4	0.482
**Ni**	49	27.1	23.0	35	32.9	29.0	14	12.5	12.0	*0.000001*
**Pb**	49	37.7	29.0	35	44.0	36.0	14	21.7	22.0	*0.001*
**Rb**	49	17.3	15.0	35	17.1	16.0	14	17.6	14.5	0.824
**Sb**	49	0.7	0.6	35	0.9	0.8	14	0.4	0.5	*0.0003*
**Sr**	41	34.9	31.0	31	36.8	32.0	10	29.0	26.5	0.254
**Ti**	49	586	600	35	683	600	14	343	350	*0.0004*
**U**	49	0.4	0.4	35	0.4	0.4	14	0.4	0.3	0.438
**V**	49	37.1	35.0	35	44.0	39.0	14	19.7	20.0	*0.0000002*
**Zn**	49	99.2	78.0	35	113.1	100.0	14	64.6	65.0	*0.0003*

**Table 2:** Spearman correlation matrix of variables measured at the lichen samples from the Syracusan petrochemical complex. The significant cases are indicated in italics. Upper critical value: 0.29.

	Al	As	Ba	Co	Cr	Cu	Fe	Mn	Mo	Ni	Pb	Rb	Sb	Sr	Ti	U	V	Zn
**Al**	1.00	*0.55*	*0.58*	−0.08	0.29	*0.32*	*0.59*	*0.39*	0.13	*0.48*	*0.42*	−0.22	*0.49*	*0.58*	*0.91*	−0.06	*0.56*	*0.50*
**As**		1.00	*0.39*	0.28	*0.52*	*0.47*	*0.68*	*0.46*	0.13	0.12	*0.42*	0.11	*0.47*	*0.35*	*0.38*	0.26	*0.38*	*0.44*
**Ba**			1.00	*0.72*	*0.65*	0.25	*0.55*	*0.59*	*0.38*	0.14	*0.44*	*0.32*	*0.39*	*0.65*	*0.45*	0.14	0.28	*0.38*
**Co**				1.00	*0.75*	*0.41*	*0.43*	*0.58*	0.13	−0.25	0.24	*0.69*	0.04	*0.62*	−0.17	*0.33*	−0.07	0.12
**Cr**					1.00	*0.47*	*0.66*	*0.64*	0.15	0.10	*0.46*	*0.56*	*0.41*	*0.71*	0.19	*0.42*	0.29	*0.40*
**Cu**						1.00	*0.41*	*0.71*	0.06	0.08	*0.55*	*0.31*	*0.40*	*0.47*	0.16	0.13	0.29	*0.37*
**Fe**							1.00	*0.63*	0.11	0.21	*0.57*	*0.22*	*0.53*	*0.53*	*0.49*	*0.32*	*0.40*	*0.58*
**Mn**								1.00	0.20	0.06	*0.60*	*0.45*	*0.33*	*0.67*	0.27	0.13	0.28	*0.41*
**Mo**									1.00	0.22	*0.35*	−0.02	0.27	0.01	0.05	−0.25	0.28	*0.45*
**Ni**										1.00	*0.42*	−0.18	*0.59*	0.10	*0.52*	−0.07	*0.87*	*0.55*
**Pb**											1.00	0.22	*0.74*	*0.55*	0.25	0.21	*0.59*	*0.68*
**Rb**												1.00	−0.04	*0.36*	−0.23	0.14	−0.03	0.00
**Sb**													1.00	*0.43*	*0.42*	0.23	*0.67*	*0.85*
**Sr**														1.00	*0.49*	0.20	0.19	*0.43*
**Ti**															1.00	−0.07	*0.50*	*0.44*
**U**																1.00	0.00	0.19
**V**																	1.00	*0.61*
**Zn**																		1.00

**Table 3:** Factor loadings (Varimax rotation) for the lichen samples from the Syracusan petrochemical complex (*p* < 0.05).

	Factor 1	Factor 2	Factor 3	Communalities
	*Anthropic*	*Anthropic*	*Geogenic*	
**Al**	0.28	0.19	***0.90***	0.90
**As**	−0.03	***0.63***	0.52	0.61
**Cr**	0.23	***0.85***	−0.02	0.60
**Fe**	0.28	***0.65***	0.53	0.72
**Mn**	0.02	***0.81***	0.25	0.67
**Ni**	***0.86***	−0.15	0.31	0.88
**Pb**	***0.68***	0.39	0.01	0.54
**Sb**	***0.80***	0.37	0.08	0.80
**Ti**	0.21	0.08	***0.92***	0.87
**V**	***0.87***	0.02	0.29	0.89
**Zn**	***0.84***	0.28	0.21	0.80
**Expl.Var**	3.55	2.64	2.50	
**Prp.Totl**	0.32	0.24	0.23	

**Table 4:** Contamination Factor (CF), Ecological Risk Index (ERI), and Pollution Load Index (PLI) of selected trace elements.

	As	Cr	Cu	Ni	Pb	Sb	V	Zn	
**CF**	1.39	1.38	1.21	2.63	2.03	1.94	2.23	1.75	
**ERI**	13.88	2.75	6.05	13.2	10.1		4.47	1.75	
**PLI**									**1.76**

## Data Availability

The data presented in this study are available on request from the corresponding author.
